# Examining the Predictive Validity of NIH Peer Review Scores

**DOI:** 10.1371/journal.pone.0126938

**Published:** 2015-06-03

**Authors:** Mark D. Lindner, Richard K. Nakamura

**Affiliations:** Center for Scientific Review, National Institutes of Health, 6701 Rockledge Dr., Bethesda, Maryland, United States of America; University of Illinois-Chicago, UNITED STATES

## Abstract

The predictive validity of peer review at the National Institutes of Health (NIH) has not yet been demonstrated empirically. It might be assumed that the most efficient and expedient test of the predictive validity of NIH peer review would be an examination of the correlation between percentile scores from peer review and bibliometric indices of the publications produced from funded projects. The present study used a large dataset to examine the rationale for such a study, to determine if it would satisfy the requirements for a test of predictive validity. The results show significant restriction of range in the applications selected for funding. Furthermore, those few applications that are funded with slightly worse peer review scores are not selected at random or representative of other applications in the same range. The funding institutes also negotiate with applicants to address issues identified during peer review. Therefore, the peer review scores assigned to the submitted applications, especially for those few funded applications with slightly worse peer review scores, do not reflect the changed and improved projects that are eventually funded. In addition, citation metrics by themselves are not valid or appropriate measures of scientific impact. The use of bibliometric indices on their own to measure scientific impact would likely increase the inefficiencies and problems with replicability already largely attributed to the current over-emphasis on bibliometric indices. Therefore, retrospective analyses of the correlation between percentile scores from peer review and bibliometric indices of the publications resulting from funded grant applications are not valid tests of the predictive validity of peer review at the NIH.

## Introduction

The National Institutes of Health (NIH) supports the training, development and research of more than 300,000 full-time scientists working throughout the United States, which stimulates economic activity, produces new businesses and products, and advances basic and clinical biomedical research [1,2]. The NIH has supported the majority of landmark studies that led to Nobel prizes [3], and more than 67% of all citations in scientific articles refer to studies funded primarily by the NIH [4]. In addition, every $1 of NIH funding produces $2.2 – $2.6 or more in economic activity [1,2,5], more than half of all the studies cited in patents filed in the biomedical field were funded primarily by the NIH [4,6,7], and many of the new drugs and biologics produced are based on research funded by the NIH [8,9], all of which contribute to significant increases in the length and quality of life [10,11].

Since the end of WWII, the NIH has largely relied on a peer review process to allocate extramural funds [12]: scientists submit applications for funding, and other scientists who are active in the same fields—their peers—are recruited by the NIH to review those applications and determine the quality, scientific merit and potential impact of the research described in those applications. Despite clear historical evidence that NIH-funded research has produced a wide range of significant and beneficial effects, the scientific community is expressing increasing criticism of the peer review process that the NIH relies on to allocate funds [13–15], and in fact, the predictive validity of peer review has not yet been empirically demonstrated [16].

Perhaps the most efficient and expedient test of the predictive validity of NIH peer review would be a retrospective analysis of the correlation between percentile scores from peer review as the predictor and bibliometric indices as the criteria. The percentile score is used by each institute as the primary influence when deciding which applications to fund, and scientists are evaluated by their academic institutions for their scientific impact based on bibliometric indices: numbers of publications and citations. Decisions about hiring, retention, promotion, tenure and compensation of academic scientists have been based on bibliometric indices for decades [17–30], and these readily-available quantitative bibliometric indices could easily be used to determine the impact of funded research projects.

A number of investigators have suggested that percentile scores and/or bibliometric indices are appropriate variables for determining the predictive validity of peer review [31–35], but there has not yet been a careful examination of whether retrospective studies using those variables are appropriate for the assessment of the predictive validity of peer review at NIH. Tests of the predictive validity of a screening procedure are dependent on the inclusion of all cases or a sample of cases selected at random across the full range of the population that is screened [36]. Such correlational studies are also dependent on the use of a valid criterion measure (e.g., a valid measure of scientific impact) on the same elements or cases evaluated with the screening procedure [37]. The present study was conducted to determine if retrospective analyses of funded research applications, using percentile scores from peer review as the predictor variable and bibliometric indices as criterion measures of scientific impact, satisfy the requirements for tests of predictive validity.

## Methods

The R01 is the most commonly used mechanism for funding research at the NIH. It typically supports a discrete, specified, and circumscribed project of up to 5 years duration, to be performed by applicants in an area representing their specific interests and competencies, based on the mission of the NIH (see http://grants.nih.gov/grants/funding/r01.htm). In 2007 and 2008, 45,874 new R01 applications were considered for funding by the funding institutes at NIH; peer review of 87% or 39,888 of those applications were managed by the Center for Scientific Review (CSR). Of those 39,888 applications, 1.4% were incomplete or had errors or were reviewed in meetings that did not assign percentile scores. The remaining 39,337 records from the NIH IMPAC II database were included in the dataset analyzed in the present study.

Each application is reviewed in-depth by three assigned reviewers, and the average of the preliminary scores from those assigned reviewers is used to determine which applications will be discussed by the full committees in the review meetings. Usually, about half of the applications reviewed by each committee—the applications with the better average preliminary scores—are discussed in each review meeting. For the applications discussed in the review meeting, all eligible committee members assign an overall impact score, and the average of those scores is used to calculate the percentile score. An application’s percentile score is the percentage of all applications reviewed by a study section with average overall impact scores better than or equal to that application (for a more detailed description of the review and scoring procedure see [38]).

Records for awarded applications included the percentile score, the total budget requested, the total budget committed by the funding institute at the time of the award, the requested duration of the project, the approved duration of the project, and the number of days between the review meeting and the date the award was issued. This dataset includes applications assigned to all the institutes at the NIH, representing all areas of research, including applications that were discussed and not discussed, and applications that were funded and not funded.

## Results

**Fig 1:**
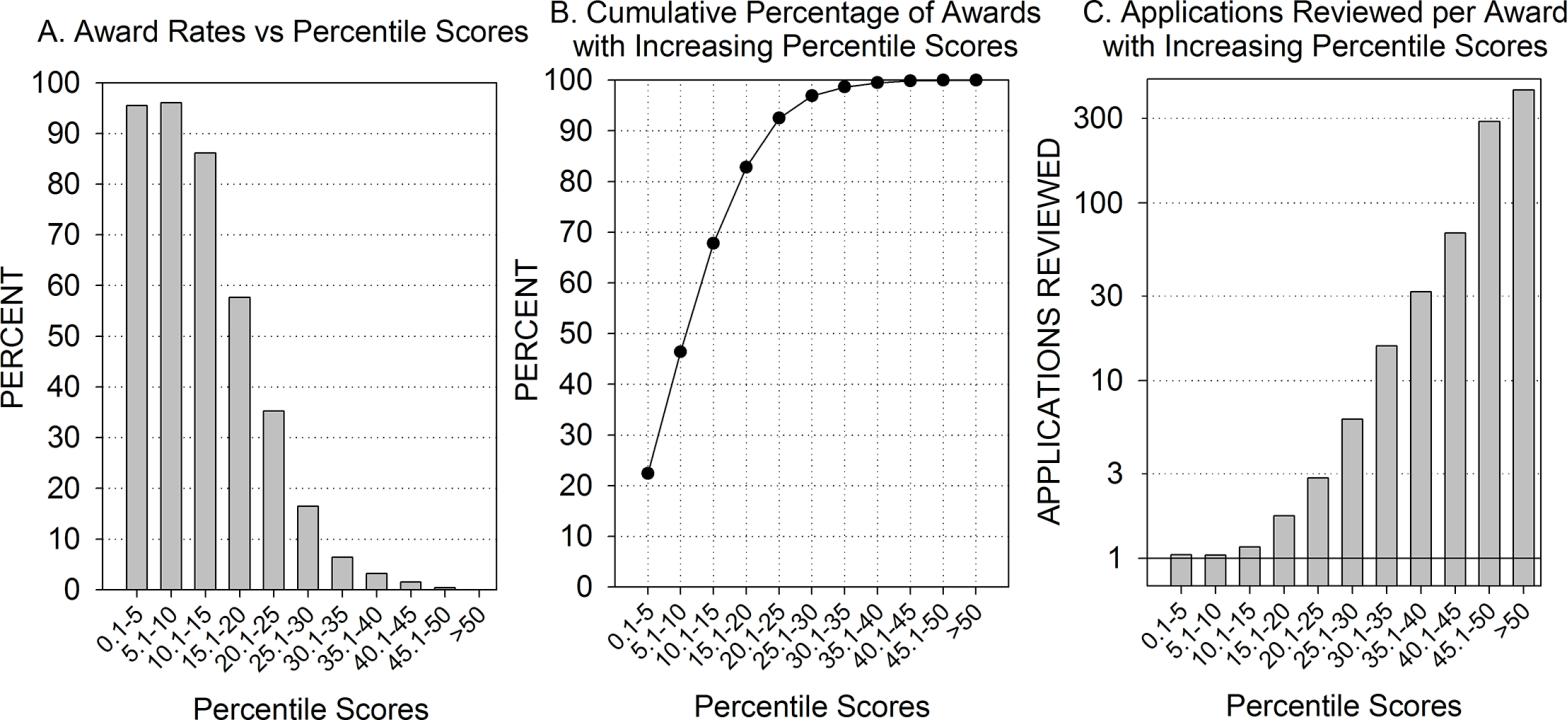
[A] Percent of applications funded decreases as peer review percentile scores increase. Approximately 95% of all applications with peer review percentile scores in the 0.1–10.0 range are funded, but only 3.2% of the applications with peer review scores in the 35.1–40 percentile range are funded. [B] Cumulative percentage of all funded applications with increasing peer review percentile scores. Almost 50% of all funded applications have peer review percentile scores in the 0.1–10 range, and 97% of all funded applications have peer review percentile scores equal to or less than 30. [C] Number of applications reviewed for each application funded increases as peer review percentiles increase. Almost every application reviewed with peer review percentile scores in the 0.1–10.0 range is funded, but only one of every 6 applications reviewed with peer review percentile scores in the 25.1–30.0 range is funded.

Overall, 48% of the applications were discussed (n = 19,049), and 17.4% (n = 6,830) were funded. As expected, most of the applications with the best percentile scores were funded, and fewer and fewer applications were funded as the percentile scores increased. For example, 95–96% of applications with scores in the top 10 percentile were funded, but only 86% of the applications in the 10.1–15 percentile range and only 57% of the applications in the 15.1–20 percentile range were funded (Fig 1A).

Among the funded applications, almost half had scores in the top 10 percentile, and 97% of all funded applications had scores in the top 30 percentile (Fig 1B). Only 3% of funded applications were beyond the 30^th^ percentile, including several at greater than the 50^th^ percentile.

Among applications in the 25.1–30 percentile range, 6 applications were reviewed for each application that was funded; for applications with percentile scores in the 30.1–35 percentile range, 16 applications were reviewed for each application funded; and for applications in the 35.1–40.0 percentile range, more than 32 applications were reviewed for each application funded (Fig 1C).

Approximately 15% of the funded applications were selected ‘out of order’. In other words, 15% of the funded applications in the present study would not have been funded if the funding decision was based purely on peer review scores, applications with better peer review scores would have been funded instead. The percentage of applications funded varies widely between the different institutes at the NIH, ranging in 2007–2008 from less than 10% at some institutes to more than 30% at other institutes, and the proportion of awards made ‘out of order’ in terms of peer review scores, also varies widely, ranging from only 3% at the National Institutes of Aging to more than 30% at some of the smallest funding institutes (see Table 1).

**Table 1:** New R01s by Funding Institute.

Institute	Institute Size (% of NIH Budget)	Award Rate 2007–2008	Awards with Percentile Scores Above Award Rates
NCI	16.4%	18%	13%
NIAID	15.1%	16%	12%
NHLBI	10.0%	19%	10%
NIGMS	6.6%	22%	17%
NIDDK	6.4%	19%	18%
NINDS	5.3%	18%	18%
NIMH	4.8%	19%	15%
NICHD	4.3%	16%	10%
NCRR	3.9%	18%	24%
NIA	3.6%	19%	3%
NIDA	3.4%	21%	14%
NIEHS	2.5%	16%	30%
NEI	2.3%	24%	22%
NIAMS	1.7%	18%	12%
NHGRI	1.7%	28%	12%
NIAAA	1.5%	24%	8%
NIDCD	1.3%	25%	21%
NIDCR	1.3%	21%	15%
NLM	1.1%	21%	20%
NIBIB	1.0%	19%	9%
NINR	0.5%	22%	30%
NCCAM	0.4%	10%	42%
FIC	0.2%	31%	21%

**Fig 2:**
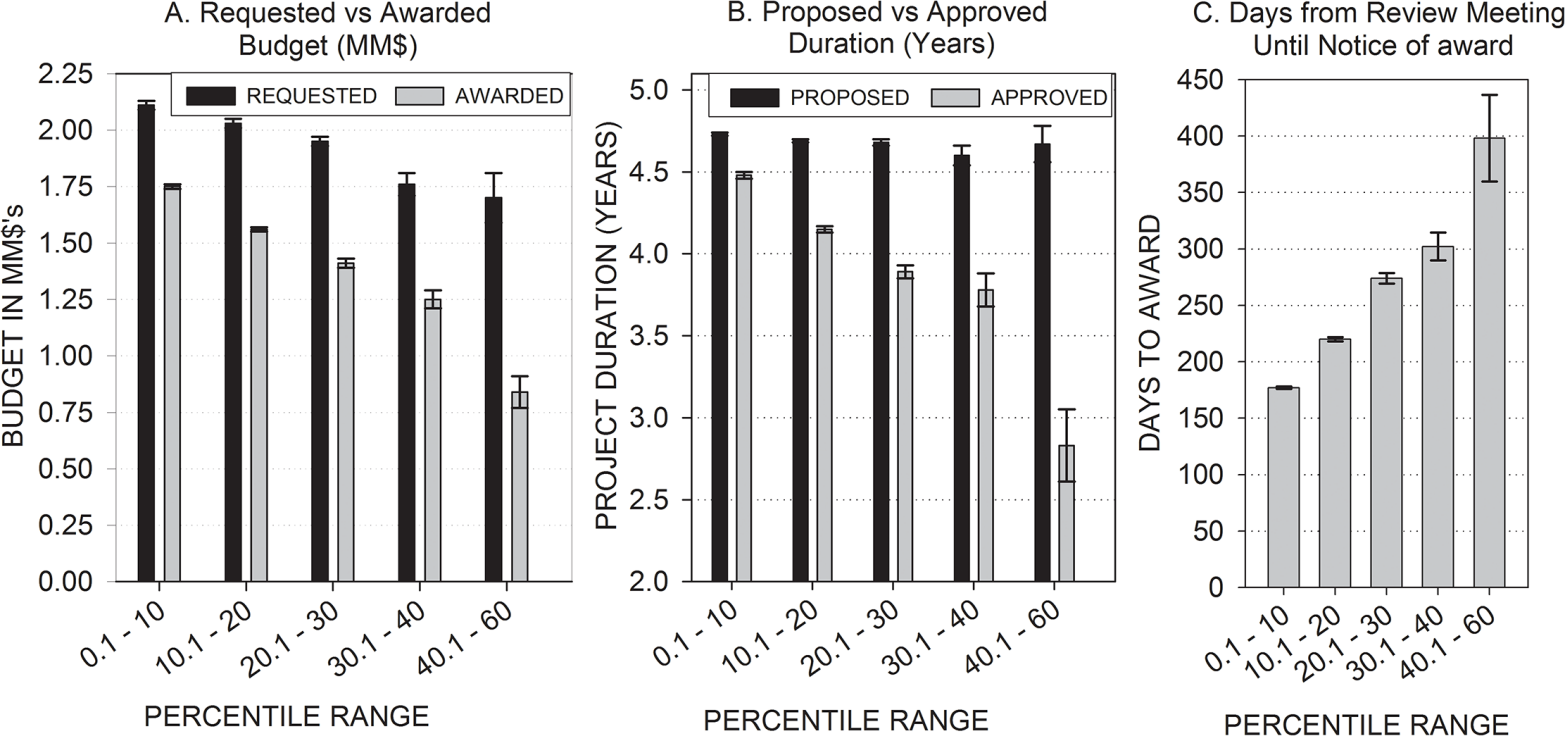
Awarded applications are classified by the range of percentile scores: 0.1–10 (N = 3,169), 10.1–20 (N = 2,486), 20.1–30 (N = 961), 30.1–40 (N = 178), and 40.1–60 (N = 36).

In addition, analyses of variance (ANOVA) of the awarded applications showed that what was funded was different from what had been initially submitted. A 5 x 2 ANOVA on the 6,830 awarded applications including percentile scores from peer review (i.e., 0.1–10, 10.1–20, etc.) and budget (i.e., requested vs. awarded) as factors in the analysis, treating budget as a repeated measure, revealed that awarded budgets were significantly smaller than requested budgets, F(1,6825) = 769.3, p < 0.0001 (Fig 2A). Furthermore, as percentile scores increased, the difference between the requested and the awarded budgets increased. The percentile score by budget interaction was statistically significant, F(4,6825) = 36.49, p < 0.0001.

A 5 x 2 ANOVA on the 6,830 awarded applications including percentile scores from peer review (i.e., 0.1–10, 10.1–20, etc.) and project duration (i.e., proposed vs. approved) as factors in the analysis, treating project duration as a repeated measure, revealed that approved project durations were significantly shorter than proposed project durations, F(1,6825) = 602.43, p < 0.0001 (Fig 2B). As percentile scores increased, the difference between the proposed and the approved project durations increased. The percentile score by duration interaction was statistically significant, F(4,6825) = 104.11, p < 0.0001.

In addition, the delay from the review meeting until the notice of award increased as the percentile scores increased, F(4,6825) = 283.05, p < 0.0001 (Fig 2C).

## Discussion

This study analyzed a large set of new (Type 1) competing R01 Research Project Grant applications reviewed at the NIH Center for Scientific Review in FY 2007 and 2008: 39,337 applications were analyzed, 48% of those applications were discussed and 17.4% were funded. Most of the applications with the best percentile scores were funded. As the percentile scores increased, fewer and fewer applications were funded and more and more applications were reviewed for each application that was funded. In addition, funded projects were different from the applications that were submitted and evaluated during peer review, and the differences between the initial peer reviewed applications and the projects that were eventually funded increased as the peer review scores increased. Furthermore, the delay from the review meeting until the notice of award increased as the percentiles increased.

The results of the present study demonstrate several reasons why retrospective studies of funded applications might fail to detect a strong linear relationship between peer review estimates of potential impact and subsequent measures of actual impact. First, tests of the predictive validity of a screening procedure are dependent on the inclusion of the full population or a sample of cases selected at random across the full range of the population that is screened. However, only a small percentage of NIH applications are funded, and those funded applications are restricted to only a portion of the entire range of applications reviewed: 97% of funded applications have peer review scores at the 30^th^ percentile or better. Such restriction of range confounds studies of predictive validity. For example, Thorndike developed a personnel screening test to determine which applicants were more likely to successfully complete pilot training school. Scores on the screening test were correlated with successful completion of pilot training at r = 0.64. However, once training was limited to the 13% of applicants with the best scores on the screening test, the restriction of range reduced the apparent correlation between test scores and successful completion of pilot training from r = 0.64 to only r = 0.18 [36].

In addition to restriction of range, the present study underlines an NIH award process in which peer review is only the first level of review at NIH, and provides evidence of the significance of the second round of review conducted by the funding institutes which consider the initial peer review scores as only a part of their funding decisions. The funding institutes at NIH identify what they feel are the best applications that most deserve to be funded. They do this by closely examining the applications and the peer review scores and written critiques. Consistent with suggestions that at least 8% of the NIH budget should be allocated to discretionary funding of high-risk, high-reward research managed by program managers at the funding institutes [39], approximately 15% of the funded applications are selected ‘out of order’ by the funding institutes. In other words, 15% of the funded applications in the present study would not have been funded if the funding decision was based purely on peer review scores, applications with better peer review scores would have been funded instead.

Especially among those applications with worse peer review scores, the funding institutes identify and ‘cherry-pick’ those few applications or parts of applications that they believe stand out from the rest of the applications in the same range, and they negotiate with applicants to address issues identified during peer review (see ‘Negotiation of Competing Award’ at http://grants.nih.gov/grants/managing_awards.htm). The extent of those negotiations and changes is reflected in the increasing differences between proposed and awarded budgets and project durations, and the increasing times from review to award. Critical information can be added, weaknesses in the design can be corrected and flawed or unnecessary experiments can be dropped.

This is not evidence of inappropriate or unethical behavior on the part of the funding institutes. They not only have the authority, they have an obligation to identify and fund the best projects. However, this means that projects are selected, revised and funded in a way that is not amenable to rigorous retrospective examination of the predictive validity of peer review scores. In addition to severe restriction of range, funded projects, especially those with worse peer review scores, are not selected at random and are not representative of other applications in the same range, and many of them are different from the projects that were initially reviewed. But the peer review scores remain unchanged: they are not revised to reflect the changes and improvements that are made during negotiations with the funding institutes. Therefore, it is not appropriate to expect that the peer review scores assigned to the original applications should be correlated with measures of the impact of the funded projects that have been revised and improved before they were eventually funded.

In addition to the issues related to peer review scores discussed above, there is also a problem with the use of citation metrics as measures of scientific impact. Citation metrics by themselves are not accepted as valid or widely used for that purpose. Reward systems shape behavior, and the current reward system based primarily on primacy of discovery and numbers of publications and citations has shaped standards of practice in ways that facilitate achievement of those objectives [25,40–45]. For example, scientists have little incentive and rarely replicate reports from other investigators [46–49] or publish results that fail to support their own hypotheses [50–55].

Papers reporting novel, robust and statistically significant effects are more likely to be published in high-impact journals and to be more highly cited, and in order to produce novel, robust, statistically significant effects, scientists often approach their research as advocates, intent on producing and publishing results that confirm and support their hypotheses [56–60] without including adequate methodological controls to prevent their unconscious biases from affecting their results [61–65]. Scientists also often conduct a large number of small studies and use exploratory analytical techniques that virtually ensure the identification of robust, novel phenomena and hypotheses but fail to report the use of exploratory, post hoc analyses or conduct the appropriate confirmatory studies [66–71].

In addition, scientists select other papers to cite based primarily on their rhetorical utility, to persuade their readers of the value and integrity of their own work: papers are not selected for citation primarily based on their relevance or validity [72,73]. Even the father of the Science Citation Index (SCI), Eugene Garfield, noted that citations reflect the ‘utility’ of the source, not their scientific elegance, quality or impact [74]. Authors cite only a small fraction of relevant sources [75,76], and studies reporting robust, statistically significant results that support the author’s agenda have greater utility and are cited much more often than equally relevant studies that report small or non-statistically-significant effects [75–81].

Given the emphasis on primacy of discovery and bibliometric indices, these strategies are clearly beneficial for individual scientists and do not constitute research misconduct; but, it is becoming more and more widely recognized that they produce problems of reproducibility of scientific findings and are therefore a source of waste and inefficiency [82]. Replication studies are rarely conducted [46–49], valid but small or non-statistically significant results are often not detected [83,84] or published [50–55], unnecessary studies are conducted because previous research results have not been published or cited [85,86], and uncontrolled biases lead to the publication of a large number of false positives [65,70,87–89]. In the vast majority of publications reporting novel phenomena, the effects are exaggerated or invalid [48–50,90–93], and high citation numbers do not provide assurance of the quality or the validity of the results [94,95]. Even studies reporting robust effects and cited more than 1,000 times are often not valid or reproducible [96,97].

Moreover, the evidence suggests that the magnitude of this problem is growing. With more and more highly qualified scientists, the ‘publish or perish’ culture continues to become ever more competitive, demanding larger and larger numbers of publications and citations in order to be hired, retained, promoted and well-compensated [98–104]. Clearly, further increasing the emphasis on the numbers of publications and citations produced by funded applications would not increase productivity, but would likely increase the problems already largely attributed to the current over-emphasis on bibliometric indices.

Instead, there is a growing consensus that scientific progress and productivity can best be increased by providing incentives to increase the integrity of the scientific literature, largely in ways that will reduce the number of publications produced and the proportion of studies reporting robust, novel effects that tend to be most highly cited [82,86,105,106]. Suggested changes to standards of practice include conducting full literature reviews and meta-analyses, citing all relevant studies, not just those that support the author’s rationale; conducting power analyses and using adequate sample sizes to detect expected effects; including and clearly communicating quality controls to prevent bias from affecting the results; clearly distinguishing between planned analyses and post-hoc exploratory analyses; and publishing results of studies in a timely manner, even if the results fail to support the investigator’s hypotheses or detect any statistically significant effect. In response, the leadership at NIH has initiated changes in the NIH peer review process to incentivize quality and reproducibility over quantity [107], and one estimate suggests that such changes could significantly increase scientific productivity [82].

## Conclusions

An appropriate test of the predictive validity of the peer review process has not yet been conducted. Such a test would need to include funded projects selected at random across the entire range of applications, and the projects would have to be conducted without changes or improvements based on issues identified during the peer review process. The impact of those applications would also have to be based on measures that appropriately value the impact and validity of the results. Citation numbers alone are not appropriate for that purpose, in part because citation numbers are often higher for studies that report exaggerated or invalid results [90,91,108]. Further increasing the emphasis on the numbers of publications and citations produced by funded applications, as some studies have suggested [32,34], would exacerbate the waste and inefficiencies already attributed to the current over-emphasis on bibliometric indices. Instead, the leadership at NIH has initiated changes in the NIH peer review process to incentivize quality and reproducibility over quantity [107], and one estimate suggests that such changes could significantly increase scientific productivity [82].
