# Correction: Examining the Predictive Validity of NIH Peer Review Scores

**DOI:** 10.1371/journal.pone.0132202

**Published:** 2015-06-29

**Authors:** The PLOS ONE Staff

## Notice of Republication

This article was republished on June 15, 2015, to replace Fig 1, which was incorrect, and to remove confidential or copyrighted material. The publisher apologizes for the errors. Please download this article again to view the correct version.
